# A Transition Zone Showing Highly Discontinuous or Alternating Levels of Stem Cell and Proliferation Markers Characterizes the Development of *PTEN*-Haploinsufficient Colorectal Cancer

**DOI:** 10.1371/journal.pone.0131108

**Published:** 2015-06-22

**Authors:** Kevin J. Arvai, Ya-Hsuan Hsu, Lobin A. Lee, Dan Jones

**Affiliations:** 1 Department of Pathology, Quest Diagnostics Nichols Institute, Chantilly, Virginia, United States of America; 2 Department of Cytogenetics, Quest Diagnostics Nichols Institute, Chantilly, Virginia, United States of America; University of Nebraska Medical Center, UNITED STATES

## Abstract

**Background:**

Stepwise acquisition of oncogene mutations and deletion/inactivation of tumor suppressor genes characterize the development of colorectal cancer (CRC). These genetic events interact with discrete morphologic transitions from hyperplastic mucosa to adenomatous areas, followed by in situ malignant transformation and finally invasive carcinoma. The goal of this study was to identify tissue markers of the adenoma-carcinoma morphogenetic transitions in CRC.

**Methods and Findings:**

We analyzed the patterns of expression of growth regulatory and stem cell markers across these distinct morphologic transition zones in 735 primary CRC tumors. In 202 cases with preserved adenoma-adenocarcinoma transition, we identified, in 37.1% of cases, a zone of adenomatous epithelium, located immediately adjacent to the invasive component, that showed rapidly alternating intraglandular stretches of PTEN+ and PTEN- epithelium. This zone exactly overlapped with similar alternating expression of Ki-67 and inversely with the transforming growth factor-beta (TGF-β) growth regulator SMAD4. These zones also show parallel alternating levels and/or subcellular localization of multiple cancer stem/progenitor cell (CSC) markers, including β-catenin/CTNNB1, ALDH1, and CD44. PTEN was always re-expressed in the invasive tumor in these cases, unlike those with complete loss of PTEN expression. Genomic microarray analysis of CRC with prominent CSC-like expansions demonstrated a high frequency of *PTEN* genomic deletion/haploinsufficiency in tumors with CSC-like transition zones (62.5%) but not in tumors with downregulated but non-alternating PTEN expression (14.3%). There were no significant differences in the levels of *KRAS* mutation or *CTNNB1* mutation in CSC-like tumors as compared to unselected CRC cases.

**Conclusions:**

In conclusion, we have identified a distinctive CSC-like pre-invasive transition zone in *PTEN*-haploinsufficient CRC that shows convergent on-off regulation of the PTEN/AKT, TGF-β/SMAD and Wnt/β-catenin pathways. This bottleneck-like zone is usually followed by the emergence of invasive tumors with intact PTEN expression but dysregulated TP53 and uniformly high proliferation rates.

## Introduction

Regulated growth of the epithelium in the normal colon is driven by crypt-localized intestinal stem cells (ISC), which alternate between quiescent and proliferative states [1]. Shifts in the subcellular localization of the Wnt-associated beta (β)-catenin (*CTNNB1*) complex and signaling through the transforming growth factor (TGF)-beta and PTEN/PIK3CA/AKT pathways have been shown to influence ISC cycling [2,3].

A phenotypically and functionally similar population of cancer stem cells (CSC) has been postulated to occur in colorectal cancer (CRC) and other tumors [4,5]. CSCs have been postulated to emerge in CRC at the earliest stages of transformation [6], possibly correlating with change described as aberrant crypt foci [7]. However, later stages of tumor progression in CRC have been more frequently modeled as stepwise transitions from hyperplastic mucosal changes to adenomatous areas (Ad), then in situ malignant transformation and finally invasive adenocarcinoma (ACA) [8,9]. In this schema, increasing proliferation rates and genetic instability lead to progressively more dysregulated and growth factor-independent growth. To date, reconciling the discontinuous model of proliferation and quiescence that underlies the CSC model with the incremental evolutionary model of the hyperplasia-adenoma-carcinoma series remains problematic [10].

Here, we show, using a large series of primary CRC cases, that in well-oriented tissue sections, many colon tumors show a clearly distinct zone of rapidly alternating proliferative and hypoproliferative colonic epithelium where numerous ISC/CSC markers are also modulated in parallel. This transition zone, which is likely overgrown by the invasive component in some tumors, highlights a critical zone of altered signaling at the invasive boundary in CRC.

## Methods

### Ethics statement

The study was reviewed and approved by the Western institutional review board (IRB) under protocol 2014–003, renewed 12/31/2014. Clinical samples utilized were excess/discarded samples that were anonymized prior to inclusion in the study with no identifying patient information retained and deemed exempt with waiver of informed consent.

### Case selection and immunohistochemistry

Cases include primary CRC cases submitted to Quest Diagnostics Nichols Institute for molecular or immunophenotyping analysis between November 2011 and November 2013. Cases included were a random selection of primary colon tumors with adequate formalin-fixed paraffin-embedded (FFPE) tissue sections. The features of morphologic transitions were recorded using hematoxylin and eosin (HE) stained slides for each case: from normal to hyperplastic mucosa, hyperplastic to adenomatous change, and adenomatous change to in situ and invasive adenocarcinoma.

Immunohistochemistry was performed on 4-μm FFPE sections using the Bond Max III (Leica Microsystems, Wetzlar, Germany), Ultra Benchmark (Ventana, Tucson, AZ), and Link (Dako, Carpinteria, CA) automated staining platforms, with epitope retrieval performed on-instrument for Leica (Epitope Retrieval Solution 2, pH 9.0 buffer) and Ventana (Cell Conditioner 1, pH 9.0 buffer) and offline for the Dako platform (TRS, pH 9.0 buffer for 40 minutes). The antibody clones and working dilutions are listed in S1 Table. The specificity of the PTEN (6H2) and SMAD4 (B-4) antibody clones utilized has been previously extensively validated in tissues and transfectants using Western blot and other techniques [11, 12]. PTEN antibody specificity was further demonstrated by absent expression by Western blot analysis of cell lysates and FFPE sections of *PTEN*-null cell lines CEM and Jurkat [13]. A peroxidase block was applied to all slides prior to antibody application. Primary antibody incubations were at room temperature for 12–32 minutes, with detection using 3,3-diaminobenzidine (DAB) (including Bond Polymer Refine for Leica, UltraView DAB for Ventana and Envision for Dako). Slides were counterstained with hematoxylin and post-counterstained with bluing.

Immunostains were scored semi-quantitatively by two of the authors (KA, DJ), with images captured using the Aperio XT slide scanner to facilitate image overlays and to assess subcellular stain localization. For Ki-67, the patterns and numbers of cells with strong nuclear positivity were recorded. PTEN staining was scored as uniform/normal in tumor; down-regulated in tumor compared to adjacent normal colon; complete/zonal loss of staining in all or part of the tumors; or as having a multifocal alternating pattern with areas of loss juxtaposed next to tumor cells with a normal pattern. For other markers, including ALDH1, β-catenin, CD44, EZH2, MGMT P53, p-SMAD (1/5/8), and SMAD4, staining intensity was assessed across the tumor in relation to non-neoplastic epithelium and/or admixed lymphocytes, as well as the subcellular localization (nuclear, cytoplasmic, membrane or combinations).

### Fluorescent *in situ* hybridization (FISH)

Glass slides containing 4-μm FFPE sections were baked at 56 °C overnight then dewaxed and rehydrated using xylene and ethanol. Slides were treated using 0.2 N HCL, formalin, pretreatment buffer wash, and protease prior to adding the FISH probe. Samples were then co-denatured at 72 °C for 5 minutes and allowed to hybridize overnight (14–18 hours) in a humidity chamber set to 37 °C. Slides were probed using a CL *PTEN/GRID1* 3-color deletion probe (MetaSystems, D-5971-100-TC). The *PTEN* locus-specific probe is labeled in orange at a 315-kb region on chromosome band 10q23.3. Additional probes in the cocktail included a locus-specific probe for *GRID1* located at chromosome band 10q23.2, labeled in green, and a probe for the centromeric control region (10p11.1-q11.1) labeled in blue.

Slides were evaluated for *PTEN* deletion by counting 30 tumor cells in ACA and Ad-ACA transition zone (if available) and comparing the number of *PTEN* signals to each of the two control regions on the *PTEN/GRID1* probe (i.e., 2R2G2B for normal, R2G2B for single loss, and 2G2B for biallelic loss). A cutoff value for *PTEN* deletion of 10% of cells was established by determining the mean false-positive rate plus 3-standard deviation using 10 cases with normal colon epithelium [14]. To localize the Ad-ACA junctional areas, FISH images were overlapped with IHC and/or HE images to mark the Ad-ACA transition. These results were compared with the regions showing loss of PTEN expression.

### Oligonucleotide/single-nucleotide polymorphism (SNP) array

After macrodissection of FFPE tumor sections, genomic DNA was extracted using the QiaAmp DNA FFPE Tissue Kit (Qiagen, Valencia, CA) and assessed using the CytoScan HD 2.6 million-probe microarray platform (Affymetrix, Santa Clara, CA) according to the manufacturer’s instructions. Up to 1 μg DNA was digested with the restriction enzyme Nsp I, ligated to adaptors, and amplified with PCR. The product was then purified using a magnetic separation technique, fragmented, and labeled before hybridization to the microarray. Sample quality was assessed at the PCR purification step. Five samples with prominent CSC-like Ad-ACA transition regions were macro-dissected and analyzed independently from the more deeply invasive ACA.

Results were analyzed using Chromosome Analysis Suite (ChAS) software (Affymetrix). For comparisons of frequency of IHC patterns and abnormal loci by oligonucleotide/single-nucleotide polymorphism array (OSA) in CSC-like cases with cases with intact PTEN, p-values were generated using Fisher’s exact test.

### Mutation analysis

Mutation analysis of genomic DNA extracted from macrodissected tumor in FFPE sections was performed for the exon 3 hotspot of *CTNNB1* and *KRAS* (codons 12, 13 and 61) by pyrosequencing, with an approximate sensitivity of 2% to 5%. In 11 cases with prominent CSC-like transition zones, Ion sequencing was performed for mutation hotspots in 34 cancer-associated genes, which additionally included *PTEN*, *SMAD4*, and *TP53*, using the Ampliseq Cancer array (Life Technologies, Carlsbad, CA). The protocol was per manufacturer’s instructions, with DNA sequencing performed on the Ion PGM platform and data analyzed using SequencePilot software (JSI MedSystems, Germany).

## Results

### Alternating proliferation highlights the Ad-ACA transition in a subset of CRC

**Fig 1:**
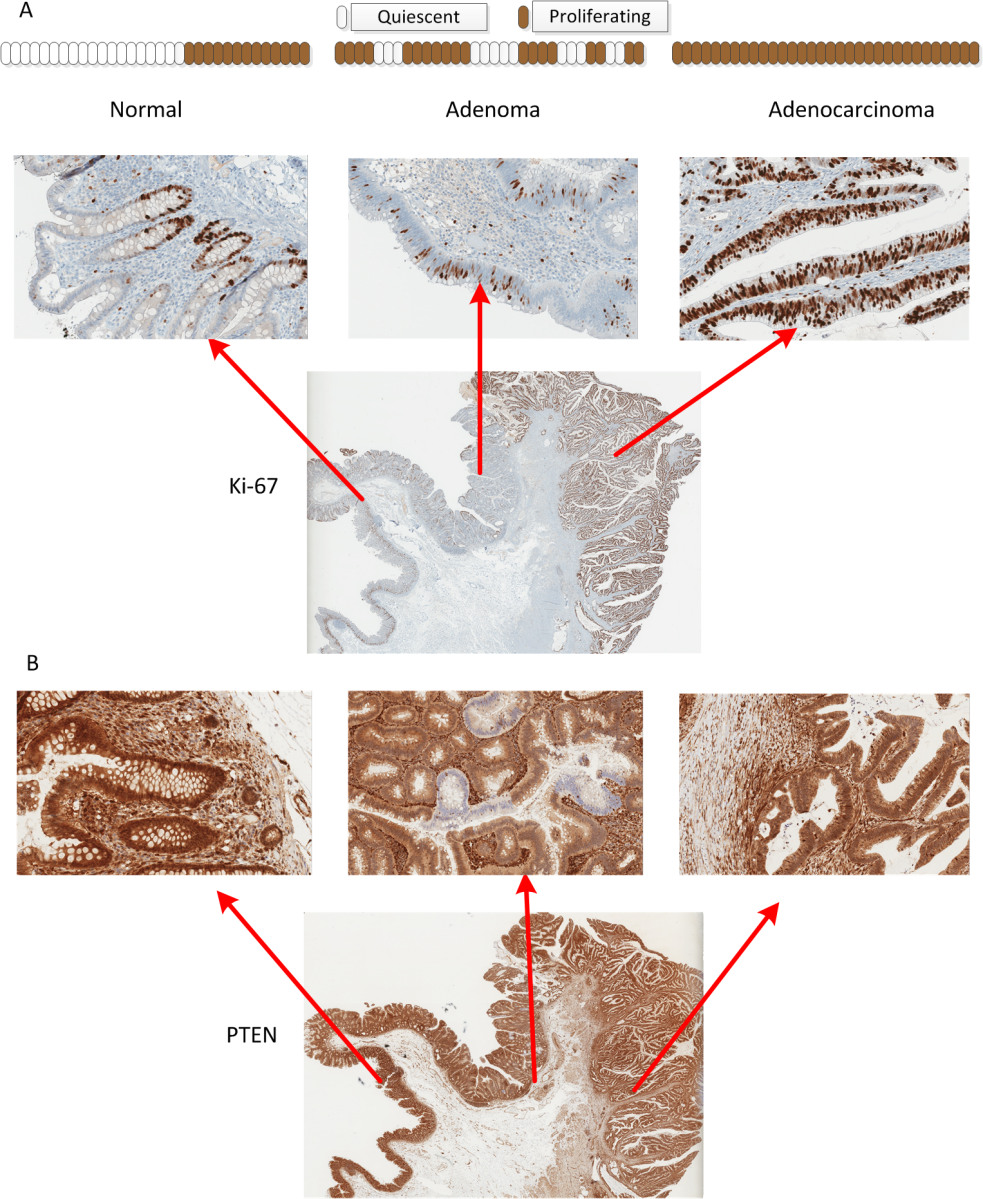
An alternating pattern of Ki-67 and PTEN at the adenoma (Ad)-adenocarcinoma (ACA) transition. (A) Ki-67 immunohistochemical staining of a primary colon cancer section shows proliferating cells restricted to the crypts in normal/hyperplastic epithelium (left); strecthes of alternating Ki-67+ proliferating and Ki-67- quiescent cells in the adenomatous region adjacent to the first areas of invasive tumor (middle); and uniform proliferation in the invasive carcinoma (right). (B) PTEN immunostaining from the same tumor shows uniform PTEN expression in the normal/hyperplastic epithelium (left), and in most of the adenomatous epithelium and invasive tumor (right). An area within the Ad-ACA transitional zone shows on-off alternating PTEN expression overlapping with the on/off Ki-67 expression.

**Fig 2:**
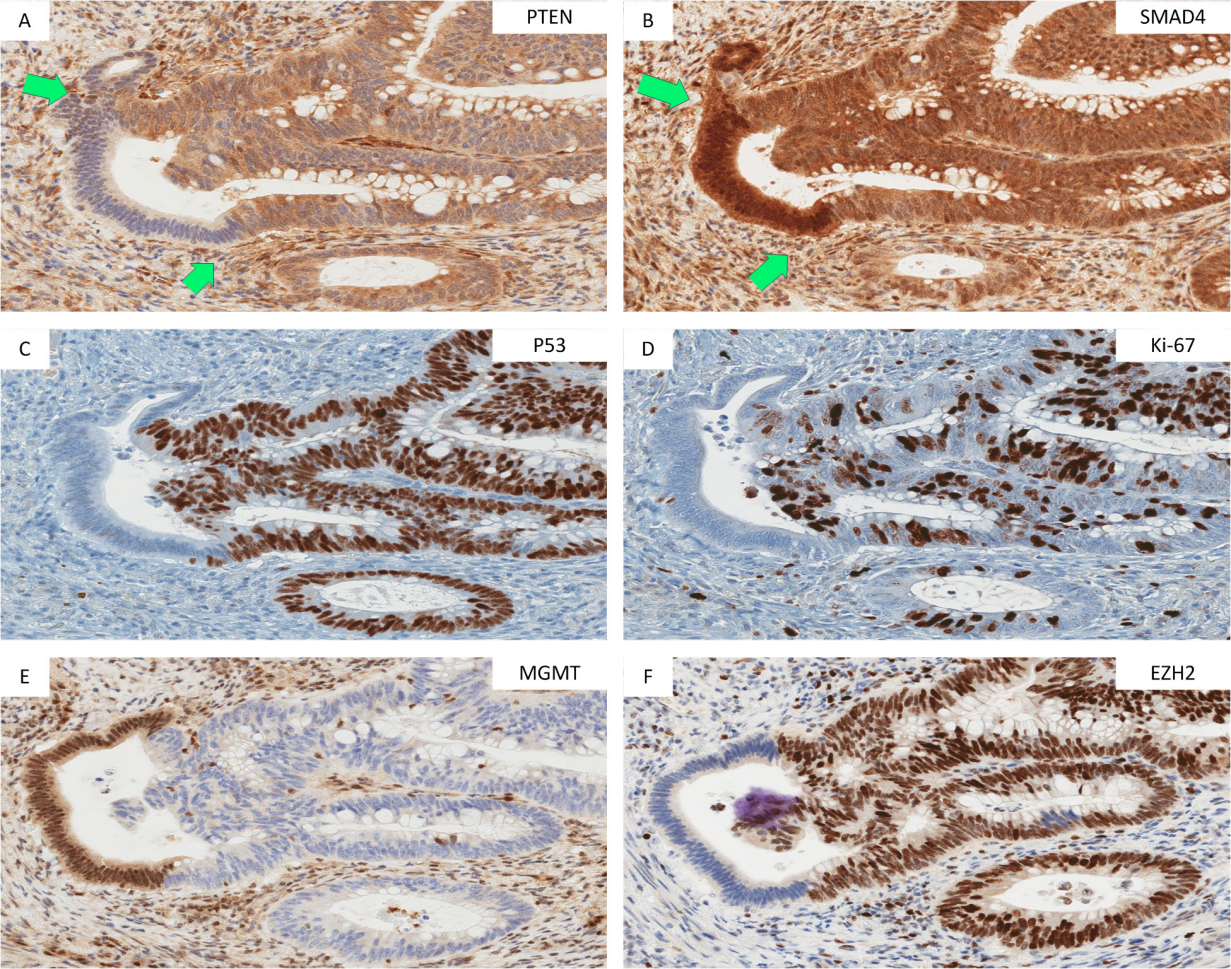
Synchronous modulation of proliferation and cancer stem cell marker expression focally in the Ad-ACA transtional zone. Immunostaining in the Ad-ACA transition zones of a primary CRC tumor showing focal loss of PTEN, Ki-67, P53, and EZH2 in the same portion of the gland that shows upregulation of SMAD4 and MGMT. The stretch of cells between the two green arrows (A & B) highlights an exact overlay of PTEN loss and SMAD4 upregulation.

We examined the expression pattern of a variety of proliferation markers across the normal-hyperplastic-Ad-ACA morphologic transitions in well-oriented FFPE tumor sections of 735 primary CRC cases. In a subset of tumors, we identified a localized zone that showed striking intraglandular alteration between Ki-67+ proliferative and Ki-67- hypoproliferative stretches of adenomatous epithelium in an area adjacent to invasive carcinoma. These zones of discontinuous Ki-67 expression occurred over a stretch of ten to several hundred tumor cells (**Fig 1A**). Despite the striking alternating IHC pattern, Ki-67+ and Ki-67 stretches were largely morphologically indistinguishable from each other and were not present at the normal/hyperplastic epithelial transition (**Fig 1A**) or in the invasive carcinoma (**Fig 1A**). In a subset of 4 cases examined, we also noted parallel discontinuous expression of multiple cell cycle regulators, including cyclin D1, MYC and P53, in these Ad-ACA transition zones (**Fig 2**)

Similar to Ki-67, the growth regulator PTEN demonstrated similar and overlapping abrupt intragland alterations in protein levels, with highly discrete/discontinuous borders (**Fig 1B**). This on/off Ad-ACA transitional zone, as detected by PTEN IHC, was prominent in 50 of the 735 (6.8%) cases studied, but was at least focally present in 75 of the 202 (37.1%) cases with intact Ad-ACA junctions (**Table 1**). This was not observed in 20 conventional colonic adenomas without associated invasive carcinoma (not shown).

**Table 1:** Patterns of PTEN IHC Expression in Primary Colorectal Carcinomas.

PTEN IHC Expression Pattern	All CRC cases (n = 735)	CRC cases with intact Ad-ACA transition (n = 202)	P Value
**Normal**	277 (37.7%)	66 (32.7%)	
**Down Regulated**	275 (37.4%)	57 (28.2%)	
**Complete Loss**	89 (12.1%)	4 (2.0%^)^	< 0.0001^#^
**Alternating On-Off Pattern**	94 (12.8%)	75 (37.1%)	< 0.0001*

^#^Comparing the frequency of preserved adenomatous areas in tumors with complete PTEN loss cases as compared to other PTEN expression patterns.

*Association of alternating phenotype with the presence as opposed to the absence of preserved adenomatous epithelium adjacent to the invasive tumor.

This alternating PTEN pattern was distinct from both the downregulated (but still uniformly expressed) PTEN IHC pattern seen in 275 cases (37.4%) and the complete PTEN loss pattern seen in 89 (12.1%) (**Table 1**). The specific pattern of PTEN expression was not significantly correlated with tumor stage, available in 108 cases. In the 75 cases with alternating PTEN and an evaluable Ad-ACA transition, the adjacent invasive ACA showed re-expression of PTEN in the invasive areas in all cases (**Fig 1B, bottom panel**).

Tumors with abrupt and uniform loss of PTEN expression by IHC had no identifiable Ad-ACA junctions in 85 of 89 cases (95.5%); the absence of residual adenomatous component was highly associated with tumors that showed complete PTEN loss (p < .0001). Tumors with complete PTEN loss of expression also lacked a preceding alternating on/off pattern of PTEN expression within the tumor or adjacent tissues in all but 2 cases.

In tumors with alternating PTEN, SMAD4, a mediator of TGF-beta signaling, also showed on-off protein modulation in these same areas in 15 of 21 cases (71.4%) but with a pattern inverse to PTEN (**Fig 2A and 2B**). PTEN-SMAD4 double-immunostaining confirmed this largely inverse relationship between the SMAD4 and PTEN staining levels (not shown). Other components of the TGF-beta pathway, including pSMAD1/5/8 and pSMAD2/3 did not reveal similar on/off expression within the Ad-ACA transition zones (**S1 Fig**).

### Pattern of cancer stem cell marker expression at the Ad-ACA transition

**Fig 3:**
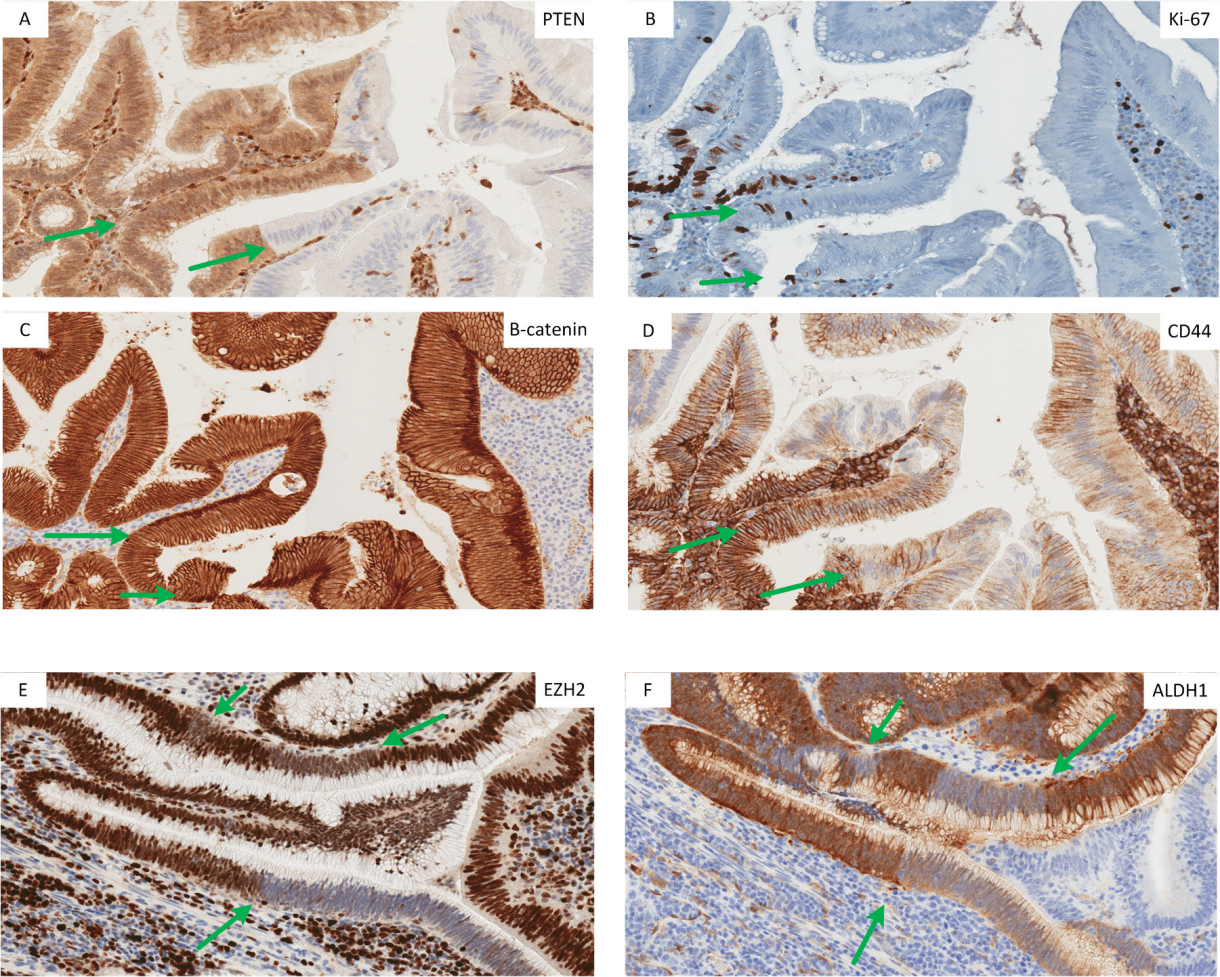
Mulitfocal alternating proliferation and cancer stem cell marker expression in a tumor with a prominent CSC-like Ad-ACA transtional zone. (A-D) Adenomatous epithelium adjacent to the invasive tumor shows prominent multifocal zones of PTEN loss and decreased Ki-67, with nuclear localization of β -catenin and decreased levels and basal membrane relocalization of CD44. (E,F) The Ad-ACA transition in another tumor shows multifocal stretches of adenomatous epithelium with loss of EZH2 and ALDH1 expression. The green arrows highlight areas where out-of-phase alternating patterns of CSC-like markers are occurring.

Given the role of PTEN/AKT signaling in mediating ISC cycling [4,15,16], we examined the level and subcellular localization of CSC markers in 21 CRC cases with PTEN/SMAD4/Ki-67 alternating transition zones. These same proliferative-hypoproliferative zones also showed dramatic discontinuity in the expression of ALDH1, EZH2, and MGMT, as well as levels and localization of CD44, and shifts in cytoplasmic/membrane and nuclear localization of β-catenin (**Figs 2 and 3**). In the stretches of cells with low-to-absent PTEN and Ki-67 positivity, β-catenin was shifted to the nucleus (**Fig 3C**). Away from the Ad-ACA transition zones, β-catenin was either fully translocated to the nucleus or present in membrane/cytoplasmic locations in nearly all cells, with only scattered single cells or small clusters of cells with nuclear-localized protein. CD44 was both downregulated and redistributed to the basal epithelium border in PTEN- areas (**Fig 3D**). MGMT was upregulated in areas that showed downregulation/absence of EZH2 and ALDH1 staining (**Fig 2E and 2F) (Fig 3E and 3F**).

Although two or more of the stem cell markers showed alternating expression in all 21 PTEN/Ki-67 alternating cases tested, the exact location of on-off marker expression for the stem cell markers was more variable which may be related to relative levels or stabilities of the proteins involved (e.g. **Fig 3** as compared to **Fig 2**). Accounting for this variability, Ki-67+ proliferative zones showed a PTEN+ P53+ EZH2^low^ ADLH1-MGMT^high^ SMAD4- immunophenotype with the inverse immunophenotype present in the Ki-67- quiescent zones.

### Correlation of *PTEN* genomic status with PTEN expression pattern

In CRC cases with prominent CSC-like transition zones, we examined the genomic findings by microarray (**Table 2**) and the mutational findings by sequencing panels, including for the exon 3 mutation hotspot in *CTNNB1* as well as *KRAS*, *PTEN*, *SMAD4*, and *TP53*. Analysis was performed in macrodissected tumor within the invasive areas.

**Table 2:** Genomic Findings by OSA in Primary CRC: Comparison of tumors with prominent CSC-like transitions compared to those with intact uniform PTEN expression.

Chromosome Locus Altered	% Altered in PTEN Loss or Prominent CSC-like Group	% Altered in Uniform PTEN Expression Group	p-value*	Type	Genes in interval related to CRC
**5q12.2q12.3**	42% (10/24)	44% (7/16)	ns	Loss	
**8p23.2p22**	42% (10/24)	50% (8/16)	ns	Loss	*CSMD1*, *CTSB*
**10q23.31**	63% (15/24)	6% (1/16)	**0.0007**	Loss	*PTEN*
**13q12.11q34**	42% (10/24)	63% (10/16)	ns	Gain	*CDX2*, *FLT1*, *PDS5B*, *STARD13*, *GAS6*
**15q14q15.2**	42% (10/24)	38% (6/16)	ns	Loss	*THBS1*, *BUB1B*, *RAD51*
**15q24.1q25.1**	46% (11/24)	31 (5/16)	ns	Loss	*NEIL1*
**17p13.2p13.1**	50% (12/24)	50% (8/16)	ns	Loss	*TP53*, *AURKB*
**18p11.32p11.31**	54% (13/24)	63% (10/16)	ns	Loss	*YES1*, *EMILIN2*
**18q11.2q12.1**	58% (14/24)	56% (9/16)	ns	Loss	
**18q12.1q22.3**	63% (15/24)	63% (10/16)	ns	Loss	*SMAD2*, *SMAD4*, *DCC*, *SMAD7*
**20p11.21p11.1**	45.8% (11/24)	50% (8/16)	ns	Gain	
**20q11.21q13.33**	62.5% (15/24)	63% (10/16)	ns	Gain	*ID1*, *BCL2L1*, *MMP9*, *AURKA*, *BMP7*

*p-values were calculated using two-tailed Fisher's exact test. Using a 2x2 contingency table, variables compared were genomic locus altered or genomic locus normal in the PTEN IHC loss/CSC group versus the PTEN normal/downregulated group; ns: not significant.

**Fig 4:**
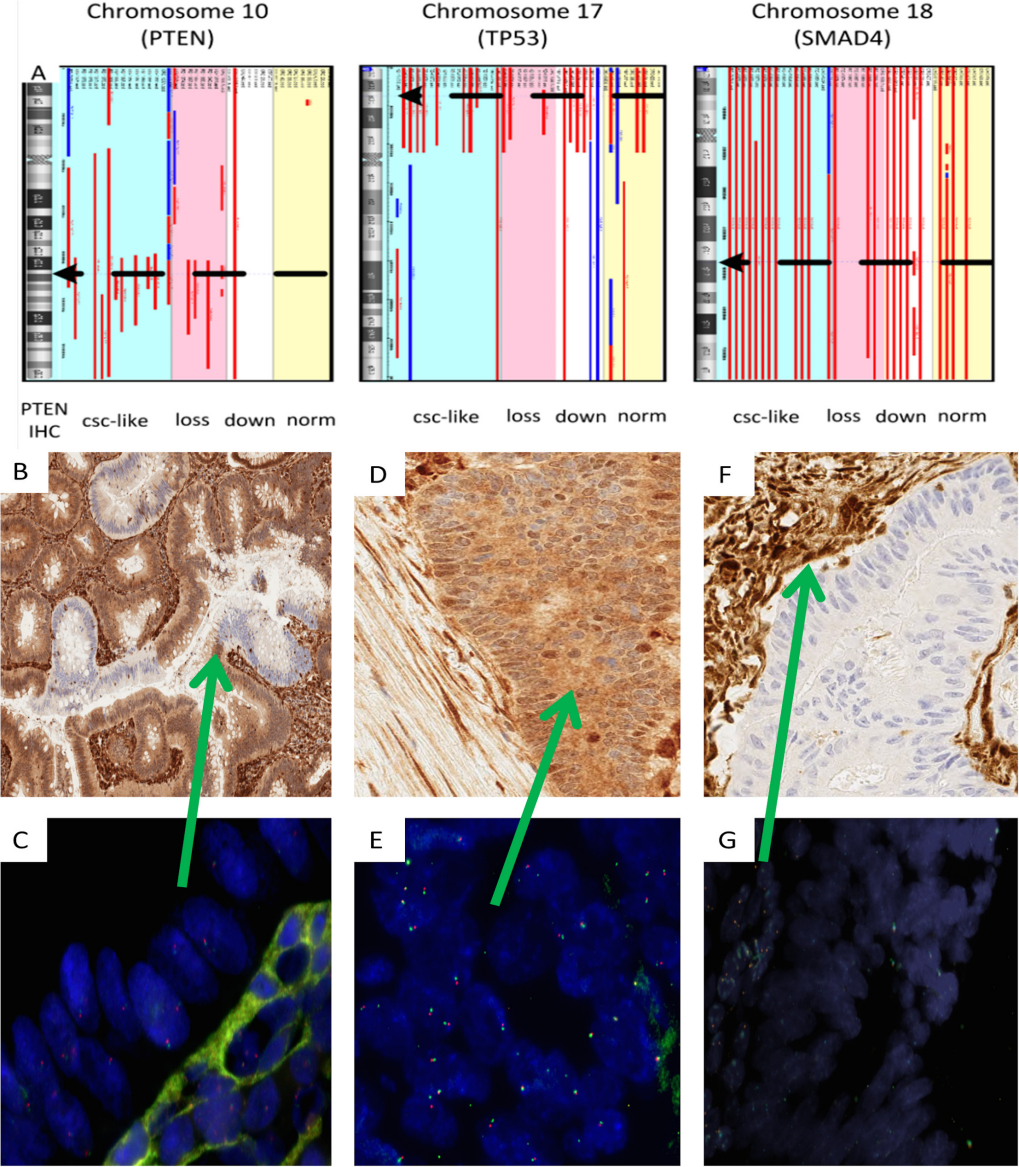
*PTEN* deletion demonstrated by microarray and FISH in CSC-like tumors. (A) Oligonucleotide/SNP array results show frequent deletion (red lines) of the *PTEN* locus on chromosome 10 (black dotted arrow) in CRC with prominent CSC-like transition zones (blue background) and in tumors with complete PTEN loss by IHC (pink background) as compared to those with uniform PTEN downmodulation or normal expression (white and yellow background). Cases with deletions of the p arm of chromosome 17 encompassing *TP53* (middle) and deletions of chromosome 18 (right) were commonly noted in CRC and were independent of PTEN IHC pattern. (B-E) Comparison of PTEN IHC and FISH in a CSC-like tumor (Case 1 from **S2 Table**). (B) High magnification of PTEN IHC with the typical alternating expression pattern at the transition zone. (C) The matched *PTEN* FISH image shows a heterogeneous pattern of loss of *PTEN* and chromosome 10 copy number in the tumor epithelial cells. (D) PTEN IHC in the invasive ACA away from the transition shows uniform expression. (E) FISH analysis in the invasive ACA area away from the transition show a uniform pattern of one copy *PTEN* loss. (F) PTEN IHC in a CRC case with absent PTEN staning. (G) *PTEN* FISH in a serial section of the same tumor as (F) showing homozygous *PTEN* deletion. The underlying stromal cells at the green arrow show normal *PTEN* copy number.

The frequency of genomic loss at 10q/*PTEN* as assessed by OSA (10/16; 62.5%) was much higher in CRC with prominent CSC-like expansion, as defined by the PTEN IHC pattern, than in cases with uniform PTEN expression (1/16; 6.3%, p = .0007) (**Fig 4A)**. In CSC-like cases, the frequency of *PTEN* deletion was even higher (13/17; 76.4%) when assayed by FISH. Using FISH, we also assessed the genetic complement in individual cells within the CSC-like zone itself and in the invasive component. These studies demonstrated much more variable deletion of chromosome 10 and the *PTEN* locus whereas in the invasive ACA away from the junction, the pattern of *PTEN* FISH was more uniformly heterozygous deletion (**Fig 4B–4E and S2 Table).**

The high frequency of *PTEN* deletion in CSC-like tumors was similar to those showing complete PTEN loss by IHC. In the latter group, however, levels of 10q loss by OSA in a subset of cases were consistent with biallelic deletion a finding confirmed by FISH in 5 cases (**Fig 4F**). Detectable *PTEN* mutations were uncommon in all groups.

The frequencies of genetic alterations at chromosome 18q/*SMAD4* 12/16(75%) and chromosome 17p/*TP53* 9/16 (56.3%) in CSC-like cases were similar to those in cases with other PTEN expression patterns (**Fig 4A**). *KRAS* mutations (83/178, 46.7%) and *CTNNB1* exon 3 mutations (1/46, 2.2%) were present in CSC-like cases at a similar frequency from tumors with other patterns of PTEN expression. In a subset of 7 cases with prominent CSC-like transition zones, DNA sequencing revealed the mutation of frequencies of 14.3% for *TP53* (1/7), 28.6% for *SMAD4* (2/7), and 57.1% for *APC* (4/7), indicating a possible higher *SMAD4* and lower *TP53* mutation rate than unselected CRC cases [17].

### Expression changes between the Ad-ACA junction and in the invasive tumor component

**Fig 5:**
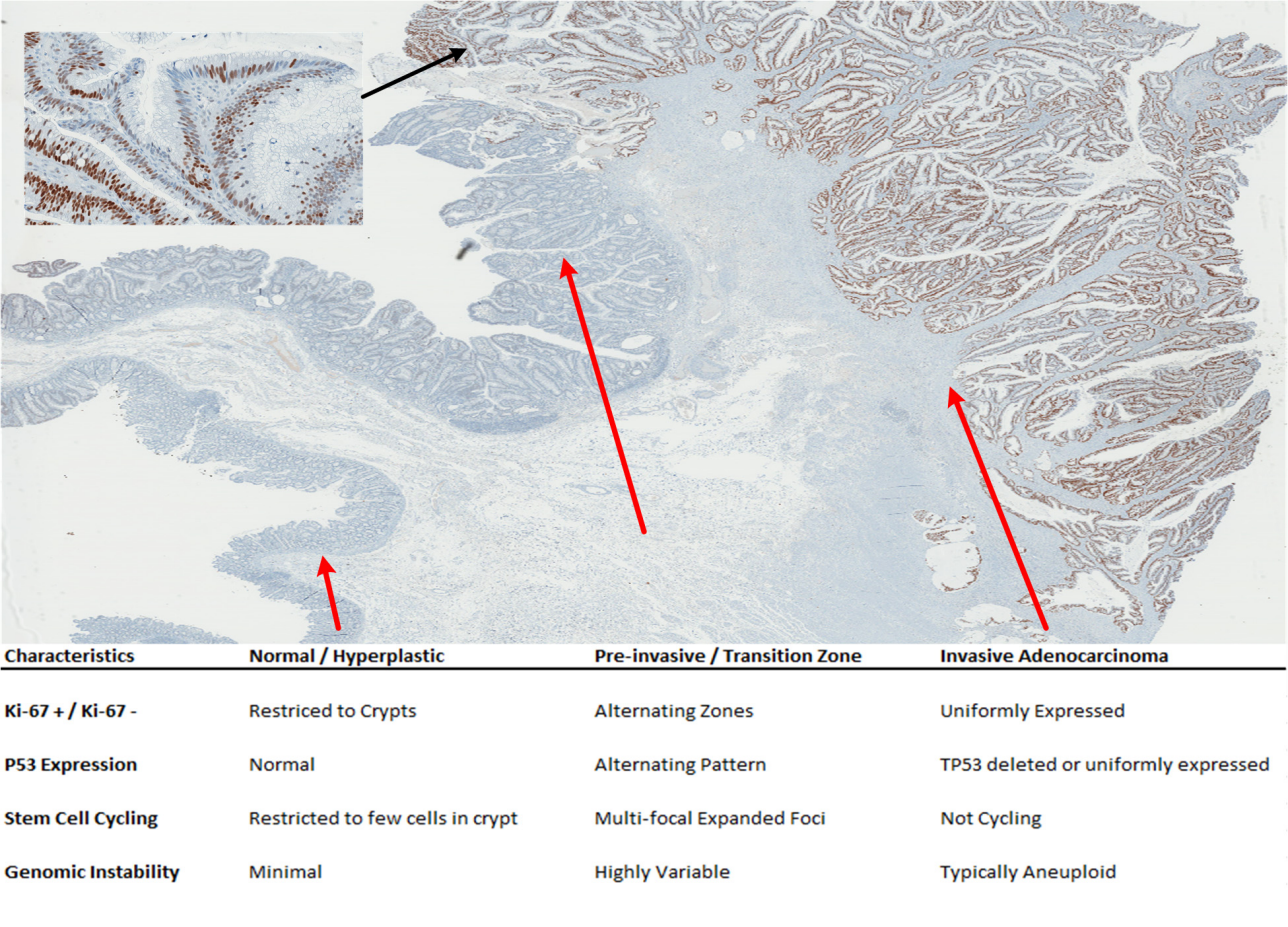
Uniform TP53 dysregulation in colon carcinoma occurring following the CSC-like transition zone. P53 IHC shows only single cell/focal expression in the normal epithelium and most adenomatous areas, alternating expression in pre-invasive/transition zone (inset) and uniformly upregulated P53 protein expression in the later stages of the invasive adenocarcinoma. This tumor is the same as the one illustrated in **Fig 1**. The common features of each morphologic transition in CRC cases with prominent CSC-like features are shown.

With the exception of MGMT, which shows more complex variable on/off expression pattern in the majority of tumors, the studied markers that were alternating within the Ad-ACA transition zone were largely uniformly expressed within the invasive ACA away from the transition. As noted above, cases with prominent alternating PTEN expression at the transition always re-expressed the protein in the invasive tumors; a similar pattern was seen for ALDH1 and EZH2. However, SMAD4 and CD44 expression was occasionally completely lost in the invasive tumor. Most striking, however, was P53 expression: TP53 expression showed a high rate of complete loss (50%) or uniform gain (41.7%) in the invasive ACA away from the Ad-ACA transition (**Fig 5**). In these tumors, the alternating P53 expression seen within the CSC-like transition zones was no longer observed.

## Discussion

By examination of the morphogenetic transition zones in a large number of primary CRC tumors, we uncovered a localized CSC-like transition zone of multifocal alternating quiescent/proliferative adenomatous epithelium affecting multiple stem cell and cell cycle markers, including Ki-67, ALDH1, β-catenin, CD44, EZH2, MGMT, MYC, PTEN, P53, and SMAD4. This transition zone, centered in the pre-invasive adenomatous epithelium adjacent to invasive tumor areas, contrasts with the more uniform proliferation and homogeneous expression profiles of the deeper invasive ACA and the adenomatous areas adjacent to non-neoplastic epithelium.

The highly significant association of *PTEN* deletion/haploinsufficiency with CRC cases that have prominent CSC-like zones implicates lower levels of the PTEN protein and consequently differential modulation of AKT kinase activity as one trigger for the CSC-like phenotype. Numerous studies in cell line and mouse genetic models have supported a role for haploinsufficiency or reduced levels of PTEN in promoting cancer progression [18–20]. Furthermore, cycling of AKT activity, due to PTEN variations or other factors such as TCL1 oscillation, is also a common finding in both cancer-related and non-neoplastic stem cell function and embryogenesis [21–24]. However, the return of PTEN to normal levels without zonal alterations in the invasive component of these CSC-like tumors implicates coordinate dysregulation of other pathways at the Ad-ACA junction. In this regard, the absence of detectable Ad/ACA junctions and the absence of a CSC-like alternating phenotype in CRC with total PTEN IHC loss indicate that the natural history of colon tumors with complete PTEN loss is distinctive [25].

Given the wide availability of both PTEN and SMAD4 immunostaining, these two markers can be used alone or in combination (including as double IHC) to complement morphologic examination in identifying the CSC-like Ad-ACA transition in limited tissue samples. Indeed, as reported for other tumors [26], PTEN IHC appears superior to gene sequencing, FISH and microarray in highlighting the dynamic PTEN regulation underlying the CSC-like transition. For both microarray and FISH analysis, in particular, PTEN functional status can be difficult to dissect given the frequent presence of aneuploidy and genetic instability in CRC. Further complicating the matter, many invasive CRC tumors are surrounded by stromal cells that may contain different, less complex, genotypes than the neighboring tumor cells. This causes reduced technical sensitivity of FFPE microarray assays. While FISH is a superior method to investigate focal genetic events, microarray was able to detect *PTEN* deletion with sufficient accuracy. Genomic analysis did reveal, however, that uniform downmodulation of PTEN levels in invasive CRC appears unrelated to *PTEN* gene deletion and unrelated to the CSC phenomenon.

Although this CSC-like zone is prominent in only a small percentage of CRC cases (**Fig 3**), it is focally present in a significant percentage of cases that show morphologically intact Ad-ACA transitions (**Fig 2**). The existence of these discrete CSC-like zones at the Ad-ACA junction in a large group of primary CRCs suggests that they may serve as a useful forerunner lesion, predicting incipient invasive transformation. In this regard, they resemble the pattern of PTEN regulation seen in glandular precursor lesions seen in endometrial cancer [27,28]. Whether these endometrial lesions display the same pattern of CSC-like proliferation/quiescence and represent a similar convergence of critical signaling pathways requires further study.

The colocalization but not perfect overlap of the on/off boundaries for many CSC markers suggests that the modulation of the proliferation/quiescent phenotype is likely quite rapid in the transition zones. These dyssynchronous boundaries seen for some markers are likely due to varying half-lives and different downregulation mechanisms for each protein, including proteolytic cleavage, transcriptional downregulation, and proteosome clearance [29,30]. Nonetheless, Ki-67+/PTEN+/MYC+/TP53+ zones alternating with Ki-67-/MGMT+/ β-catenin^nuc^/SMAD+ zones is the characteristic pattern seen in many cases.

The pattern of alternating markers observed in the transition zones favors activating and inhibitory interactions between the TGF-beta/SMAD, PI3K/Akt and Wnt/ β-catenin signaling pathways [31]. Interactions between AKT and BMP/TGF-beta signaling, as noted here for PTEN and SMAD4, are a feature of the stem cell phenotype in some models [16,32,33]. The modulation of SMAD signaling (as assessed by phospho-SMAD antibodies) and SMAD4 expression in opposition to PTEN in a number of the CSC-like cases may reflect hyperresponsiveness of the junctional epithelium to BMP/TGF-beta signaling. To help drive the cyclical proliferative/quiescent properties of the transition zone, effects of SMAD activation on inhibition of Wnt signaling [34,35] could oppose the effects of increased EZH2 on driving β-catenin activity [36].

Another possible coregulator of the CSC-like phenotype at the Ad-ACA transition is MYC, which also alternates in parallel with PTEN, Ki-67, and SMAD4. In cell line models, MYC overexpression can drive PTEN upregulation and EZH2 downmodulation by inhibiting AKT [37]. Once PTEN levels and AKT activity begin to oscillate, effects of the broader stem cell transcriptional program become likely [38]. AKT activity in the absence of PTEN has also been shown to promote genetic instability, which is observed at the CSC-like transitions and would tend to promote genetic progression [39].

In most CRC with prominent CSC-like zones TP53 dysregulation, as evidenced by uniform P53 IHC loss or gain, occurred in the invasive tumor away from the transition zone. This suggests that TP53-driven outgrowth of a dominant invasive CRC clone usually follows and is perhaps driven by genetic selection within the CSC-like transition zone. It is possible that these transition zones are a general phenomenon of the pre-invasive stage of carcinogenesis, where AKT and Wnt/ β-catenin are cyclically regulated. The fluctuating TP53 levels and highly regulated proliferative-quiescent transition suggests the zone may function as a tumor bottleneck stage preceding the emergence of more uniformly TP53-dysregulated invasive subclones.

In this study, we mostly identified the CSC-like transition in *PTEN*-haploinsufficient tumors with intact Ad-ACA junctions. Whether these transitions are present in colon tumors that follow other transforming pathway is currently unclear. However, several technical reasons could account for this detection failure: the Ad-ACA junction may be obliterated by overgrowth of the invasive component; it may not be sampled owing to incomplete sectioning of the tumor; or it could be difficult to visualize because of the complex architecture of some tumors. Additionally, for any given marker, aberrant absence or overexpression due to mutation or gene deletion can mask the transition zone. This happens frequently with SMAD4, where mutation or loss of heterozygosity can result in uniform complete loss of expression. Therefore, a wider panel of markers may be needed to detect the CSC-like Ad-ACA transition zone with the highest sensitivity in other CRC cases.

## Conclusions

In colorectal tumors with an intact adenoma-carcinoma zone, PTEN and SMAD4 frequently show an inverse alternating expression pattern in this transition. This pattern overlaps with similar alternating expression of proliferation and cancer stem cell (CSC) markers. This on-off pattern for PTEN, SMAD4 and most stem cell markers is abrogated in the invasive carcinoma away from this adenomatous junction. These results support the presence of a highly modulated and likely transitory CSC-like phenotype within the evolving carcinoma at the invasive tumor boundary. The frequent presence of heterogeneity of the copy number of *PTEN* at these junctional areas and uniform *PTEN* haploinsufficiency in most of the invasive tumors showing this finding support a central role for this gene in driving this stem cell-like phenomenon.

## Supporting Information

S1 Fig Pattern of SMAD dysregulation in colon cancer. (A) A case of colon carcinoma with alternating areas of SMAD4 expression and loss. (B) In this case, SMAD2/3 activation is intact as assessed by nuclear expression of phospho-SMAD2/3. (C) Phospho-SMAD1/5/8 antibody shows an intact pattern of activation in multiple proliferating tumor foci unrelated to alternating SMAD4 expression. Green arrows mark the SMAD4 on/off area for the other markers, which do not show parallel alterations.
(TIF)

S1 Table Antibodies used for immunohistochemistry. (XLSX)

S2 Table Comparison of PTEN expression and *PTEN* genomic status in the Ad-ACA transition and within the ACA. (XLSX)
