# Supplementary figures and images for: A Transition Zone Showing Highly Discontinuous or Alternating Levels of Stem Cell and Proliferation Markers Characterizes the Development of PTEN-Haploinsufficient Colorectal Cancer
Source: PLoS One. 2015 Jun 22;10(6):e0131108. doi: 10.1371/journal.pone.0131108 (PMC4476594; doi:10.1371/journal.pone.0131108)

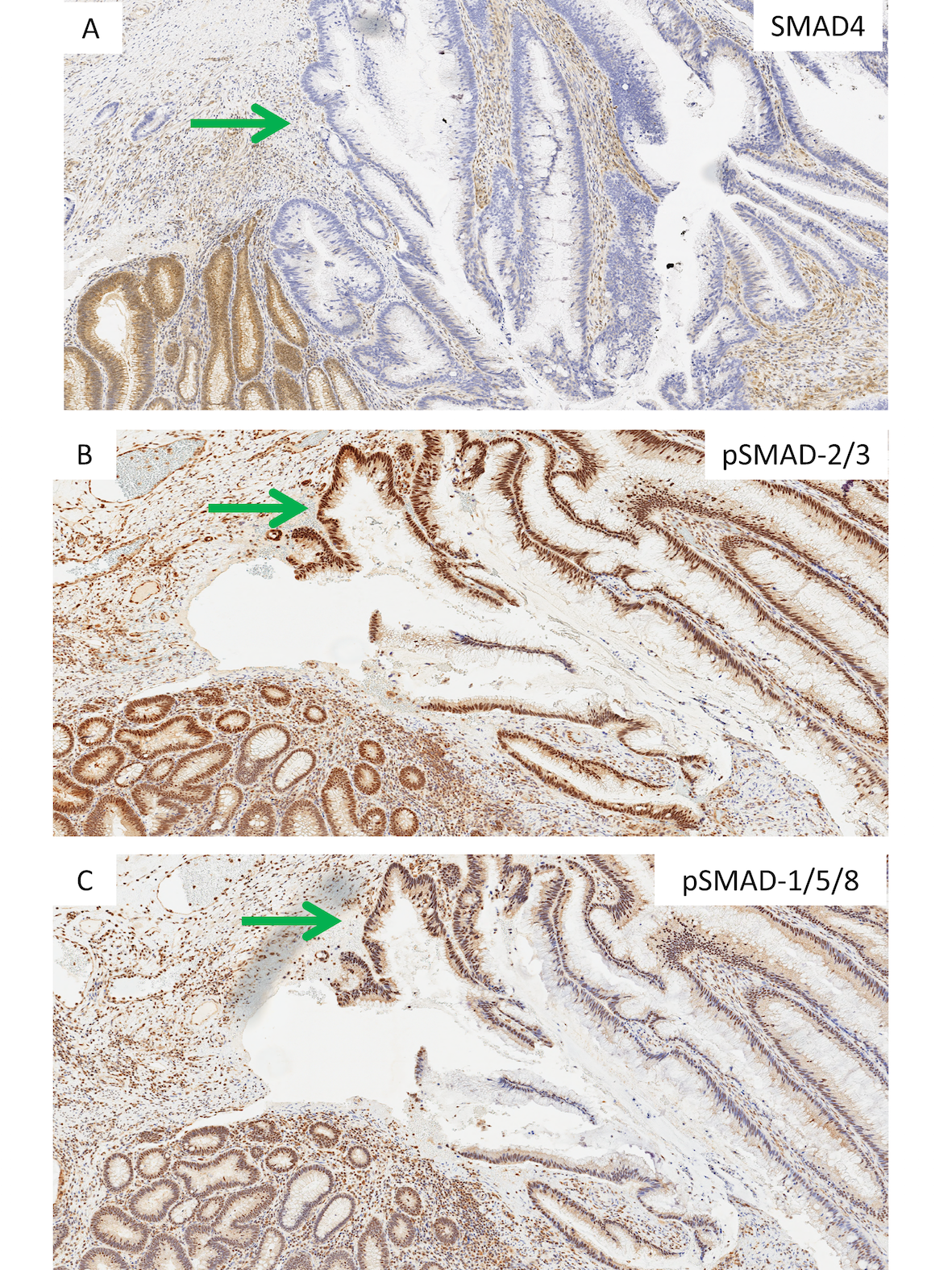

Supplement: S1 Fig — (A) A case of colon carcinoma with alternating areas of SMAD4 expression and loss. (B) In this case, SMAD2/3 activation is intact as assessed by nuclear expression of phospho-SMAD2/3. (C) Phospho-SMAD1/5/8 antibody shows an intact pattern of activation in multiple proliferating tumor foci unrelated to alternating SMAD4 expression. Green arrows mark the SMAD4 on/off area for the other markers, which do not show parallel alterations. (TIF) [file pone.0131108.s001.tif]
